# Corrosion Properties of 34CrMo4 Steel Modified by Shot Peening

**DOI:** 10.1155/2017/1928198

**Published:** 2017-12-21

**Authors:** Qiang Zheng, Kejian Li, Xueguo Yin, Bingbing Li, Chunhong Li, Yilong Ma, Jianchun Sun, Dengming Chen

**Affiliations:** ^1^School of Metallurgy and Materials Engineering, Chongqing University of Science and Technology, Chongqing 401331, China; ^2^The Center of Material Analysis and Testing, Chongqing University of Science and Technology, Chongqing 401331, China

## Abstract

A nanocrystalline layer was prepared on the surface of 34CrMo4 steel by time controlling shot peening (SP, i.e., 1, 5, 10, and 20 minutes). Field emission scanning electron microscopy (FESEM), X-ray diffraction (XRD) analysis, and transmission electron microscope (TEM) were applied to analyze the surface, cross-sections, and grain size of the specimens before and after SP. The electrochemical corrosion behavior was used to simulate a liquid under the oil and gas wells environment. It was characterized by the potentiodynamic polarization test and electrochemical impedance spectroscopy (EIS). The analysis results show that the surfaces of the SP samples were very rough and had numerous cracks. A passive film on SP surface was formed by nanocrystalline grains. However, the passive film formed in the initial stage was not dense or uniform, and cracks occurred in the passive film during peening, resulting in a decrease in corrosion resistance.

## 1. Introduction

The Cr-Mo series steels are widely used because of their excellent properties by reasonable heat treatment process, such as favorable hardenability and shock absorption, less tendency of temper brittleness, and good corrosion resistance [1, 2]. Owing to those superior performances, 34CrMo4 is one of the Cr-Mo series steel which is always used as a drill stem. In recent years, the drilling tools are vulnerable in high-sulfur and high-temperature oil and natural gas wells, leading to corrosion or even fracture failure and resulting in huge economic loss. Nanocrystalline materials [3, 4] have attracted considerable amount of interest due to their excellent performance in a multitude of areas, such as mechanical strength, wear resistance, and corrosion resistance [5–8]. Surface modification includes surface deposition, physical vapor deposition (PVD) and chemical vapor deposition (CVD) [9, 10], laser surface treatment [11, 12], and shot peening (SP) [13–16], which is an effective way to improve the surface properties of materials. Currently, SP is an economical, low cost, and effective method for industrial production, significantly improving wear resistance, fatigue resistance, and corrosion resistance of the material. The severe plastic deformation results in cold working hardening of the surface layer successfully being applied to pure metal [17], various alloys, and steels [18–20].

This study aims to improve the corrosion properties of 34CrMo4 steel upon SP; the effect of peening time on corrosion resistance was investigated in the simulated environment of liquid under oil wells. In addition, a variety of methods were used to characterize and analyze the mechanism of corrosion resistance.

## 2. Materials and Methods

Chemical composition of the 34CrMo4 steels used in the test is shown in Table 1, which is consistent with the nominal material composition. Before the SP, the specimens are heat-treated by quenching from 870°C and annealing in 560°C for 2 hr [21]. After heat treatment, specimens were ground down to 2000 grits SiC sand paper and then polished on cloth to reduce scratching. The process parameters of the SP carried out the intensities of 0.5 mm, which was produced using cast steel shots 0.6 mm in diameter, and treated times were adoptive for 1 min, 5 min, 10 min, and 20 min, respectively.

The microstructure morphology of untreated and SP samples was etched in a 3% HNO_3_ + 97% alcohol solution, followed by optical microscope (OLYMPUS, GX71) characterization. The surface before and after shot peening and the electron back-scattered diffraction (EBSD) result of raw sample were observed by scanning electron microscope (FESEM, JSM-7800F). The different depth microstructure of peening samples was researched by transmission electron microscope (TEM, JEM-2100F operating at 200 kV). Phase composition and crystallite size measurements of shot peening samples were studied by X-ray diffraction (XRD, SmartLab-9) with a CuK*α* radiation, having a working voltage of 45 kV and current of 200 mA. The electrochemical behavior of 34CrMo4 steel before and after SP was evaluated by electrochemical workstation (CH Instrument, CHI-660E), and both evaluations were performed with a three-electrode system (i.e., 34CrMo4 steel, Calomel electrode, and Pt as working electrode, reference electrode, and counter electrode, resp.). In order to better simulate the actual working environment of the 34CrMo4 steel under the oil well, the electrolyte composition consisting of 4710 mg/L NaCl, 780 mg/L KCl, 33850 mg/L CaCl_2_, and 33600 mg/L MgCl_2_ was similar to that of the drilling fluid [22].

## 3. Results and Discussion

### 3.1. Microstructure Analysis

**Figure 1:**
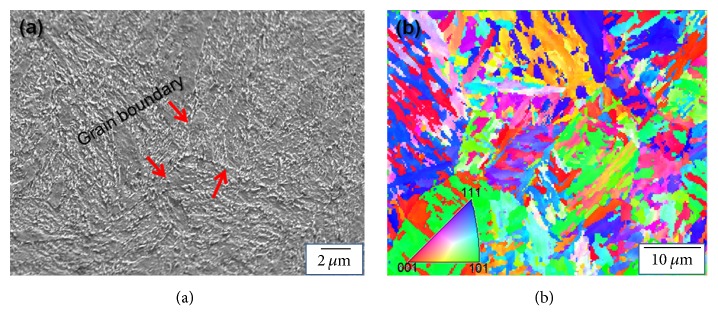
Matrix of 34CrMo4 steel (a) FESEM image and (b) EBSD IPF mapping.

The microstructure of 34CrMo4 steel matrix was investigated by FESEM and can be observed in Figure 1, which presents the FESEM image of the 34CrMo4 steel. In Figure 1(a), grain boundaries with triple junction are marked by a red arrow. Moreover, the grain size is in micrometer scale, and fine carbide particles are distributed in the matrix. In Figure 1(b), EBSD inverse pole figure (IPF) mapping indicates representative fine and homogeneous tempered martensite structure. The colors are significantly different crystal directions.

**Figure 2:**
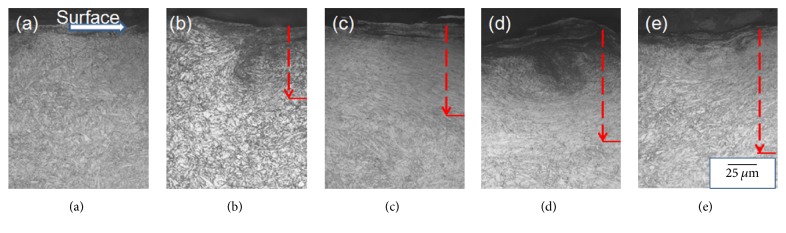
Cross-sectional OM observation of test specimens: (a) untreated, (b) 1 min peened, (c) 5 min peened, (d) 10 min peened, and (e) 20 min peened. Dashed arrows show the affected depth by shot peening.

Figure 2 shows the optical micrograph (OM) of all the cross-sectional specimens. It is the typical fine tempered martensite structure in picture (a) where the organization is overwhelmingly homogeneous. All of the deformation layers of peened specimen are clearly observable in the top layer, and the depth of strain layer, which slowly increases with the prolongation of peening time, is from 80 *μ*m to 150 *μ*m at the red arrows direction in the mircoscale.

**Figure 3:**
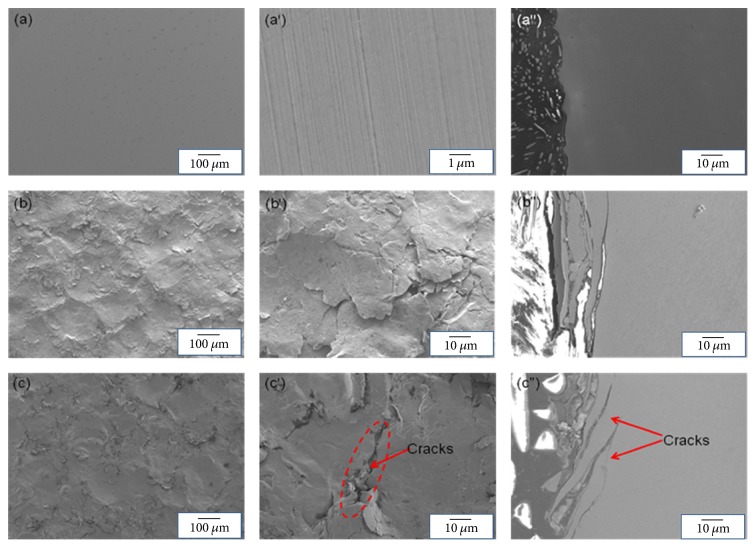
FESEM images of surface and cross-sections of the untreated (a, a′, and a′′), 5 min shot peened (b, b′, and b′′), and 20 min shot peened specimens (c, c′, and c′′).

The morphology of the surface and cross-sectional layers by SP was studied.  Figure 3 shows that SP surfaces for 5 min and 20 min are more than rough compared with the untreated surface; it can be seen that the surfaces are made up of many defects with the loose lamellar structures and small cracks under greater magnification. On the other hand, the vertical plane (cross-section) has a great deal of cracks, with length being about tens of microns and depth ranging from 10 to 30 microns. The surface condition included roughness and cracks, which had a significant effect on the material properties, especially corrosion resistance.

**Figure 4:**
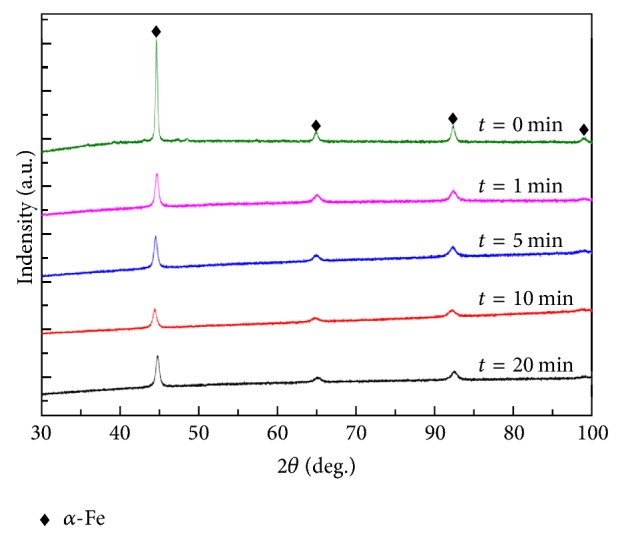
X-ray diffraction patterns of untreated and peened samples with different peening times.

Figure 4 illustrates that the XRD profiles exhibited no phase transformation of the untreated and SP samples, which crystallizes uniquely in the *α*-Fe phase (PCPDF#65-4899) [23]. However, with peening time increased to 20 min, full width half maximum (FWHM) of the peaks gradually increase, which is attributable to grain refinement in the peening process. The mean grain size of surface layer-calculated according to the Scherrer formula of the XRD data was about to ~87.2 nm for 1 min, ~55.3 nm for 5 min, ~33.8 nm for 10 min, and ~32.2 nm for 20 min of peened specimens, respectively. However, the grain size of untreated sample is approximately a micrometer.

**Figure 5:**
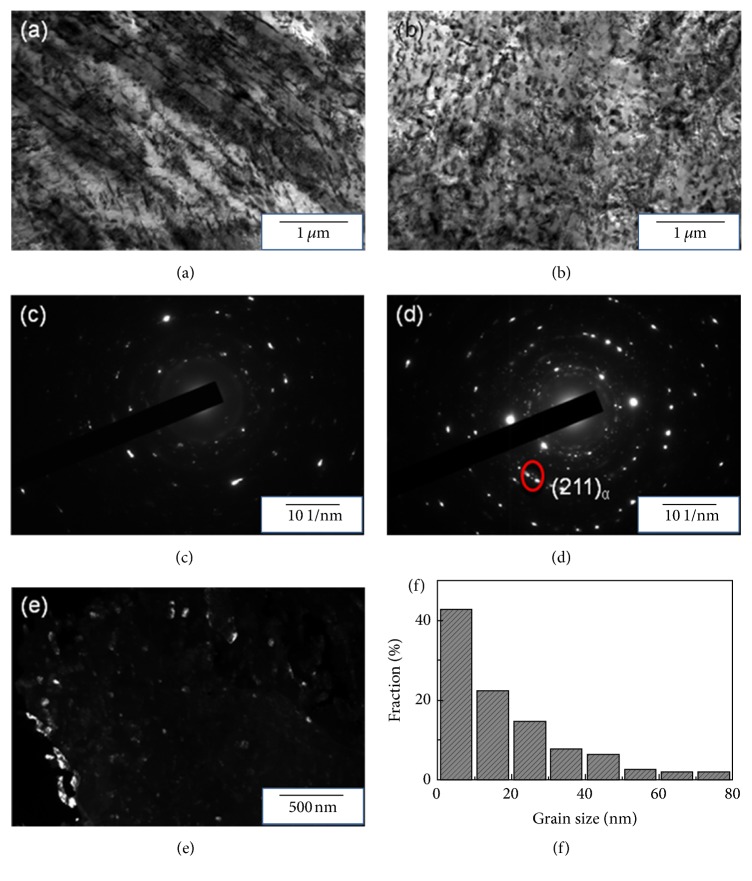
TEM images of (a) untreated and (b) 20 min SP specimens at ~10 *μ*m depth, with the corresponding (c) SADPs and (d) peened specimen. (e) TEM dark field image at [211] direction of martensite phase and (f) grain size measurement.

Figure 5 presents the TEM results: (a) illustrates a typical fine quenched and tempered martensite structure in the 34CrMo4 steel. It is apparent that a series of laths are arranged in parallel. The mean width of lath is 200~500 nm and length is 1.5~2.5 *μ*m.  Figure 5(b) displays the TEM micrograph at a depth of 10 *μ*m by 20 min peening, indicating that lath of tempered martensite has disappeared. (c, d) are SADPs taken on (a) and (b) areas, respectively. The ring-like pattern seen in (d) indicates that the martensite phase has a random orientation.  Figure 5(e) is a dark-field image corresponding to [211] diffraction direction of martensite phase, indicating that average grain size is ~30 nm (Figure 5(f)), which is identical with the results received by XRD.

### 3.2. Corrosion Properties

**Figure 6:**
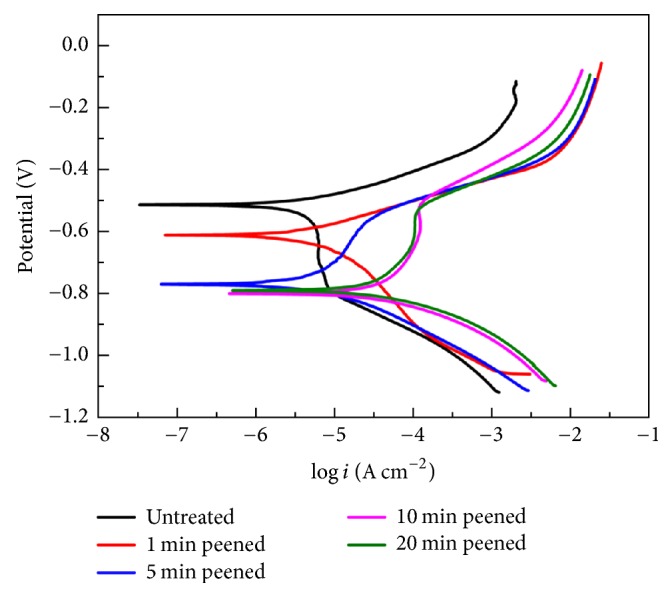
The polarization curves of untreated and SP specimens.

**Figure 7:**
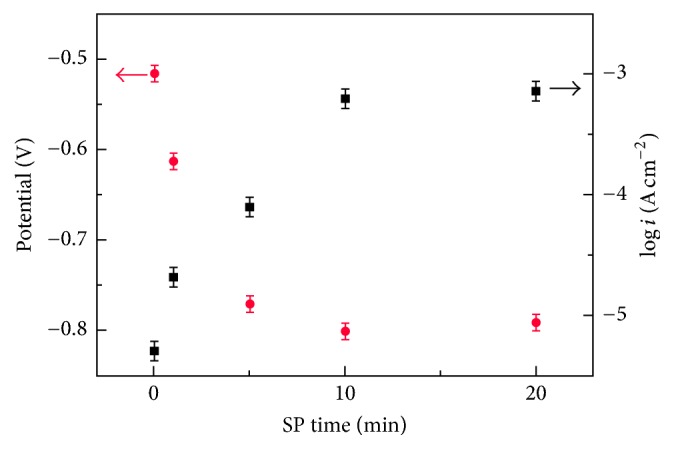
The values of *E*_corr_ and log⁡*i*_corr_ of untreated and SP specimens.

The potentiodynamic polarization curves of the 34CrMo4 steel before and after shot peening in a simulated liquid environment of the oil well are measured. The curves do not discernibly alter them for 1 min peening when compared with the untreated specimen. It is important to highlight that the anode curve exhibits a sharp slope (i.e., passivation zone) when the peening time is over 5 min, as can be observed in Figure 6. The slope on the anode curves may be a result of the grain refinement produced during the SP process.  Figure 7 displays the outcome of the potentiodynamic polarization test for all samples. It can be seen that the corrosion potential monotonically decreased with the extension of peening time, and the corrosion current density gradually increases. The peened surface is rough and has numerous defects, leading to the electrolyte being prone to gathering in the corner and moving along the cracks, as seen in Figure 3.

**Figure 8:**
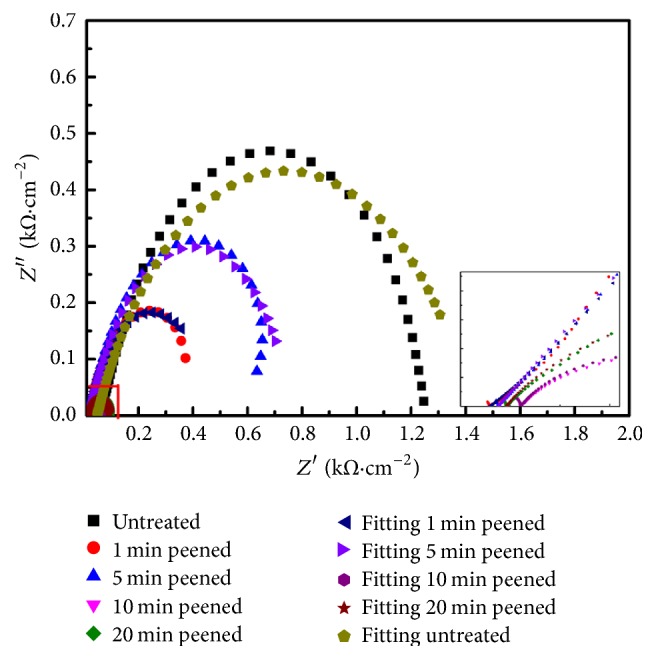
The Nyquist plots of untreated and SP specimens.

**Figure 9:**
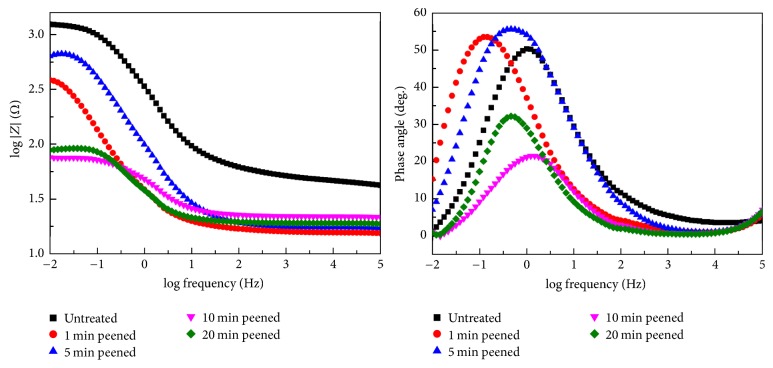
Bode plots of untreated and SP specimens.

Figures 8 and 9 show that capacitive arc radius (polarization resistance) clearly reduces through SP treatment. With careful attention it was found that the capacitive arc radius first increases and then slowly decreases. From the small photograph inserts in the Nyquist plots, it is obvious that the peened sample shows a small capacitive arc and the untreated sample does not in the high frequency range. The results are also supported by the Bode plot with the relation between frequency and phase angle, indicating the time constants exit two values.

**Figure 10:**
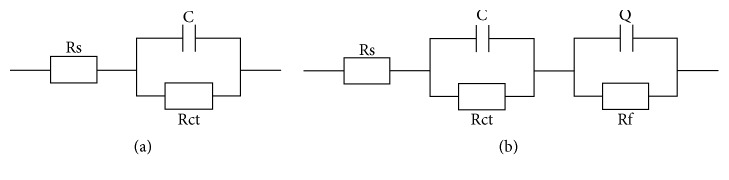
Equivalent circuits of (a) untreated and (b) SP specimens.

According to the electrochemical experimental data shown in Figure 10, two equivalent circuits are simulated. The circuit in Figure 10(a) is representative of the untreated sample, and Figure 10(b) belongs to the SP. The simulation data of the equivalent circuits of EIS curves, thereinto, contained solution resistance (Rs), electrical capacitance of double layer (C), resistance of double layer (Rct), and electrical capacitance of natural oxide film (Q, constant phase element) with the corresponding dispersion coefficient (Nn), as well as the resistance of passive film (Rf); these are presented in Table 2.

Based on the experimental results, the effect reason and mechanism of corrosion resistance of the 34CrMo4 steel by shot peening had been demonstrated. Firstly, the corrosion resistance property is a clear illustration of how the untreated and peened samples have changed. Corrosion resistance property decreased the steel shot with high energy to hit the specimen surface, bringing about numerous defects including cracks, roughness, loose lamellar structures, and others as can be seen in Figure 5. Those defects make the corrosion liquid easy to spread in the matrix interior, leading to material oxidation. The nanostructure was then produced by peening and XRD data calculations, causing the passive film on the metal surface to emerge. The corrosion resistance property is measured by potentiodynamic scanning; it is illustrated that the passivation zone is evident with increasing peening time, which is a consequence of the nanocrystalline layer of metal surface being well-distributed. On the other hand, the equivalent circuit of EIS for the peened sample shows the nature of passive film. The results indicate that the passive film is rough, which can be demonstrated by the dispersion coefficient expressing a noncapacitance. Moreover, it should be noted that the Rf value of the peened sample was found to rise first from 216 to 312.1 Ω·cm^2^ and then decreased to 20.86 Ω·cm^2^; this information is exhibited in Table 2. To analyze and explain this particular phenomenon, it can be suggested that the passive film at the beginning is not compact and uniform during short peening time. Accordingly, increasing the peening time up to 10 min or longer drives off the passive film fracture, with the final result worsening corrosion resistance. Therefore, it can be proposed that corrosion resistance can be only improved when the SP time is appropriate.

## 4. Conclusion

The 34CrMo4 steel surface was modified by SP. The following use of XRD diffraction and TEM results observed and confirmed the existence of a nanolayer, with the grain size at the top of the surface being approximately ten nanometers.

The surface of SP shows many defects, including roughness and cracks, leading to an obvious decrease in corrosion resistance of 34CrMo4 steel. The electrochemical behavior results indicated that the peened materials show greater passivation compared with the untreated material on account of crystal refinement. It can then be seen that the resistance of passive film increases at first and then decreases by the fitting results of equivalent circuits, which is attributable to the passive film at the beginning not being compact or uniform during short peening time. However, film fracture occurred after increasing peening time.

## Figures and Tables

**Table 1:** Chemical composition of the 34CrMo4 steel (wt.%).

Elem.	C	Si	P	S	Cr	Mn	Mo	Cu	Ni	Fe
Standard	0.30~0.37	0.10~0.40	≤0.035	≤0.035	0.90~1.20	0.60~0.90	0.15~0.30	≤0.030	≤0.030	Bal.
Measured	0.34	0.22	0.026	0.02	1.11	0.68	0.15	-	-	97.4

**Table 2:** Fitting results of equivalent circuit.

Peening time (min)	Rs Ω·cm^2^	C (10^−4^) F·cm^2^	Rct Ω·cm^2^	Q (10^−3^) F·cm^2^	Nn	Rf Ω·cm^2^
0	59.24	3.921	1048	-	-	-
1	15.81	2052	264.6	16.97	0.5594	216
5	17.58	56.84	418.5	4.208	0.6695	312.1
10	11.85	0.000426	10.28	6.388	0.7357	56.2
20	19.26	0.01354	53.28	13.08	0.7213	20.86
